# Genetic Characterization of Methicillin Resistant and Sensitive, Vancomycin Intermediate *Staphylococcus aureus* Strains Isolated from Different Iranian Hospitals

**DOI:** 10.5402/2012/215275

**Published:** 2012-12-20

**Authors:** Seyed Asghar Havaei, Amir Azimian, Hosein Fazeli, Mahmood Naderi, Kiarash Ghazvini, Siamak Mirab Samiee, Zahra Masoumi, Mojtaba Akbari

**Affiliations:** ^1^Department of Microbiology, School of Medicine, Isfahan University of Medical Sciences, P.O. Box 73461-8174, Isfahan, Iran; ^2^Department of Medical Biotechnology, School of Medical Sciences, Tarbiat Modares University, Tehran, Iran; ^3^Department of Molecular Biology and Genetic Engineering, Stem Cell Technology Research Center, Tehran, Iran; ^4^Department of Microbiology, School of Medicine, Mashhad University of Medical Sciences, Mashhad, Iran; ^5^Food and Drug Laboratory Research Center, Ministry of Health and Medical Education, No. 408, Emam Khomeini Avenue, Tehran, Iran; ^6^Reference Health Laboratories, Ministry of Health and Medical Education, Tehran, Iran; ^7^Department of Epidemiology, School of Medicine, Isfahan University of Medical Sciences, P.O. Box 73461-8174, Isfahan, Iran

## Abstract

*Background*. Global concerns have been raised due to upward trend of Vancomycin Intermediate *Staphylococcus aureus* (VISA) and Vancomycin Resistant *Staphylococcus aureus* (VRSA) reports which mean casting doubt on the absolute effectiveness of the last line of antibiotic treatment for *S. aureus*, vancomycin. Hence, epidemiological evaluation can improve global health care policies. *Methodology*. 171 Isolates of *Staphylococcus aureus* were collected from different types of clinical samples in selected hospitals in Isfahan, Mashhad, and Tehran, Iran. Then, they were evaluated by agar screening, disk diffusion, and MIC method to determine their resistance to vancomycin and methicillin. The isolated VISA strains were then confirmed with genetic analysis by the evaluation of *mecA* and *vanA* genes, *SCCmec, agr*, and *spa* type, and also toxin profiles. MLST was also performed. *Results and Conclusion*. Our data indicated that 67% of isolated *S. aureus* strains were resistant to methicillin. Furthermore, five isolates (2.9%) had intermediate resistance to vancomycin (VISA). In contrast to usual association of VISA with MRSA strains, we found two isolates of MSSA-VISA. Therefore, our data suggests a probable parallel growing trend of VISA towards MSSA, along with MRSA strains. However, more samples are required to confirm these primarily data. Moreover, genetic analysis of the isolated VISA strains revealed that these strains are endemic Asian clones.

## 1. Introduction

*Staphylococcus aureus* is one of the major causes of hospital and community acquired infections that can vary from mild superficial lesions to acute deep and systemic infections. Increasing antimicrobial resistance in *Staphylococcus aureus* has led to major concerns about the future of antimicrobial therapy of its infections. Finding methicillin resistant *Staphylococcus aureus* (MRSA) strains, just two years after the clinical use of betalactamase resistant betalactams, put specialists into trouble with treating these infections. About twenty years later in 1980s, alternative effective anti-Staphylococcal antibiotics such as vancomycin were introduced [1, 2]. Thence, for many years vancomycin was the drug of choice for MRSA infections, until the first *S. aureus* isolate with reduced sensitivity to vancomycin (vancomycin intermediate *Staphylococcus aureus* (VISA)) was reported in Japan in 1997 [3]. Thereafter, several reports of VISA from USA [4], France [5], Brazil [6], Korea [7], and other parts of the world [8] were published and resulted in increasing concerns about the effectiveness of vancomycin therapy in serious Staphylococcal infections. Reduced sensitivity to vancomycin in *S. aureus* occurs due to several genetic and phenotypic alterations in wild-type bacteria including altered expression of regulatory genetic elements, thickness of cell wall, changes in the penicillin binding protein (PBP) profiles, and decreased cell wall autolysis [9]. Since 2002, there have been also several reports of vancomycin resistant *Staphylococcus aureus* (VRSA) strains, mainly from the United States, India, and Iran [10–19]. The majority of the reported VISA isolates are MRSA strains with reduced susceptibility to vancomycin even though a few reports have also shown this phenomenon in methicillin sensitive *Staphylococcus aureus* (MSSA) isolates [9, 20–22]. Data obtained from genetic analysis of VISA isolates was rarely reported in Asian countries, particularly from Iran. Here, we reported genetic characteristics of five VISA strains, isolated from admitted patients in three teaching hospitals in Isfahan, Mashhad, and Tehran, Iran, in the autumn of 2011.

## 2. Methodology

171 isolates of *Staphylococcus aureus* were obtained from patients admitted between September 23 2011 and December 21 2011 at selected hospitals in Isfahan, Mashhad, and Tehran, Iran.

These isolates were taken from blood, urine, sputum, wound, abscess, nose, throat, eye, and respiratory tract samples. *S. aureus* isolates were identified by conventional biochemical tests including gram staining, catalase, mannitol fermentation, slide, and tube coagulase test and DNase.

### 2.1. Screening for Methicillin and Vancomycin Resistance

All *S. aureus* isolates were screened for oxacillin and vancomycin resistance using agar screening method. The isolates that had grown in vancomycin agar screening media were retested with phenotypic tests to confirm the identification. It is noteworthy that the data obtained from *spa* typing, considered to be highly specific for *S. aureus,* was in align with phenotypic data and confirmed the isolated strains as *S. aureus*.

### 2.2. Antimicrobial Susceptibility Test

Antibiotic susceptibility test was carried out using disk diffusion method (MAST DISKS) according to guidelines of Clinical Laboratory Standards Institute (CLSI) [23, 24]. The list of the antibiotics used in the test is presented in Table 2. Strain of *S. aureus* ATCC 25923 was used as control. 

### 2.3. MIC Determination

Oxacillin and vancomycin agar dilution was used for all *S. aureus* isolates according to guidelines of Clinical Laboratory Standards Institute (CLSI). *E*-Test method (Biomeriux Strips) was used for redetermination of MIC results for *S. aureus* strains capable of growing in the vancomycin agar screening plate and/or having vancomycin MIC 4–8 *μ*g/mL. Methicillin resistance was defined as the capability of growth in agar screening media including 4% NaCl + 6 *μ*g/mL oxacillin and MIC ≥ 4 *μ*g/mL and vancomycin intermediate resistance was defined as vancomycin MIC of 4–8 *μ*g/mL [23]. Strains of *Staphylococcus aureus* ATCC 29213 and *Enterococcus faecalis *ATCC 52199 were used as controls.

### 2.4. Genomic DNA Extraction

Genomic DNAs of *S. aureus* isolates were extracted using QIAamp DNA minikit. According to manufacturer's protocol for bacterial cells, we added lysostaphin at the final concentration of 30 *μ*g/mL in lysis buffer. 

### 2.5. PCR

PCR amplification was performed with a TAKARA Gradient PCR TP600 thermal cycler in a volume of 50 *μ*L. We used EmeraldAmp MAX PCR Master Mix (Takara, Japan) for all PCR reactions.

**(i) PCR Identification of *mecA* and *vanA* Genes**

The primers used for amplification of *mecA* and *vanA* genes are listed in Table 3. PCR was performed with the following thermal setting: 5 min at 94°C for initial enzyme activation followed by 40 cycles of amplification (denaturation at 94°C for 30 sec for *mecA* and 1 min for *vanA*; annealing at 57°C for 45 sec for *mecA* and at 55°C for 1 min for *vanA*; extension at 72°C for 30 sec for *mecA* and 2 min for *vanA*) and final extension at 72°C for 5 min.

**(ii) Multiplex PCR for Detection of Toxin Genes**

The primers used for amplification of *Panton-Valentine Leukocidin (pvl), Toxic Shock Syndrome Toxin-1 (tst1), alpha hemolysin* (*hla*), and *Enterotoxin C* (*sec*) genes are listed in Table 3. PCR was performed with the following thermal setting: 5 min at 94°C for initial enzyme activation followed by 40 cycles of amplification (denaturation at 94°C for 40 sec; annealing at 60°C for 40 sec; extension at 72°C for 1 min) and final extension at 72°C for 5 min.

**(iii) Multiplex PCR for *SCCmec* and *agr* Typing**

*SCCmec* and *agr* typing were performed as previously described [25, 26]. The primers used for the PCR are listed in Table 3.

### 2.6. MLST

Multilocus Sequence Typing was carried out by PCR and sequencing of the internal fragments of *arc*, *aro*, *glp*, *gmk*, *pta*, *tpi*, and *yqi genes of S. aureus* as previously described [27].

### 2.7. *spa* Typing

Determination of *spa* type was performed by PCR and sequencing of polymorphic X region of *spa* gene was carried out as previously described [28].

### 2.8. Nucleotide Sequencing

Amplified PCR products were purified with QIAquick Gel Extraction Kit. The purified PCR products were sequenced with an ABI 3730XL DNA analyzer (Applied Biosystems) in both directions. The sequences were used for both confirmation and sequence-based typing methods (MLST and *spa* typing).

## 3. Results

Of 171 *S. aureus* isolates analyzed, we found that 115 isolates (67%) were MRSA and 5 isolates (2.9%) showed intermediate resistance to vancomycin. The demographic data and antibiogram results of these strains are listed in Table 2. Moreover, genetic evaluation results including *mecA* and *vanA* genes PCR, *Sccmec* types, *agr* groups, toxin profiles, *spa* types and Sequence Types (STs) are presented in Table 1.

## 4. Discussion

Isolation of VISA strains has been reported in many parts of the world. Resistance to vancomycin has led to global concerns owing to the fact that vancomycin is considered as the last effective drug of choice for Staphylococcal infections [3–8]. The isolation of heteroresistant VISA (hVISA) and VISA strains has been increasingly reported from different Asian countries with various prevalence rates [3, 7, 29, 30]. There are not many reports concerning the prevalence rate of VISA strains in Iran; however, in 2008, Saderi et al. reported the mean prevalence rate of 1.8% for VISA isolated from clinical samples based on evaluation of four teaching hospitals in Tehran [31]. In our study, we found five VISA strains (2.9%) amongst 171 *S. aureus* isolates. Our data shows an upward trend in the isolation of VISA strains in Iran that might be due to the high and uncontrolled vancomycin prescription rate, even though more research is needed to elucidate the matter. 

Some reports have been published concerning the antimicrobial susceptibility patterns of VISA strains, isolated in various parts of the world [32, 33]. In most of these studies, the majority of the isolates were resistant to clindamycin but were susceptible to most of the other anti-Staphylococcal antibiotics [34–36]. Most of VISA strains in this study were also resistant to clindamycin. In contrast, Levofloxacin, Ciprofloxacin, Cotrimoxazole, and Gentamycin were effective on most of the VISA isolates (Table 2). 

The majority of the previously reported VISA strains were resistant to methicillin; however, there have been also a few reports about methicillin sensitive *Staphylococcus aureus*-VISA (MSSA-VISA) strains [9]. Two isolates of our five VISA strains, both obtained from the same hospital in Mashhad, were susceptible to methicillin; however, one of them was isolated from throat sample of a hospitalized patient in internal ward and another was isolated from wound sample of an outpatient in urgent ward that had not had any history of hospitalization. With regard to genetic characteristics, the two strains were similar; for instance, both of them belonged to *agr* group II and sequence type 8 (ST8) and therefore, clonal complex 8 (CC8). However, their *spa* types and toxin profiles were different. The wound isolated strain (VISA4) had *spa* type t008 and was positive for *Panton-Valentine Leukocidin* (*pvl*) and *alpha Hemolysin* (*hla*) genes. On the other hand, the throat isolated strain (VISA5) belonged to *spa* type t0189 and merely had *Toxic Shock Syndrome Toxin* (*tst1*) gene. Owing to the fact that sequence type is considered as a long-term and stable epidemiological marker over time and due to full homology in the sequence type of the two isolates, we deduced that they might have a common ancestor. On the other hand, alterations in some genes, such as *spa *and virulence factor genes, might be due to the fact that these elements are more prone to genetic alterations in a shorter period of time.

The relation between *agr* group, type of infection, geographical area, and resistance to some antibiotics, particularly vancomycin, is highlighted in some articles [37]. Impaired function of accessory gene regulator (*agr*) has been associated with the development of VISA strains [9]. *agr* I or II is associated with VISA strains isolated from diverse geographical regions [32, 33, 37, 38]. Our findings are in concordance with these reports. Both of our MSSA-VISA strains belonged to *agr* II but the other three MRSA-VISA strains had *agr* I. 

All the patients with MRSA-VISA either had a previous history of hospitalization or were being hospitalized at the time of sampling. Moreover, their *SCCmec* types belonged to types I and III. Collectively, the data indicates that the isolated MRSA-VISA strains were hospital-acquired MRSA (HA-MRSA). VISA1 strain was positive for all evaluated toxin genes including *pvl*, *hla*, *sec,* and *tst1* which can increase its potential pathogenicity; however, this isolate was susceptible to most of the tested antibiotic disks (Table 2). Interestingly, *pvl* gene was found in HA-MRSA “VISA1” despite the fact that it is mostly found in CA-MRSA strains. VISA 2, 3 did not have any major toxin genes. 

**Figure 1:**
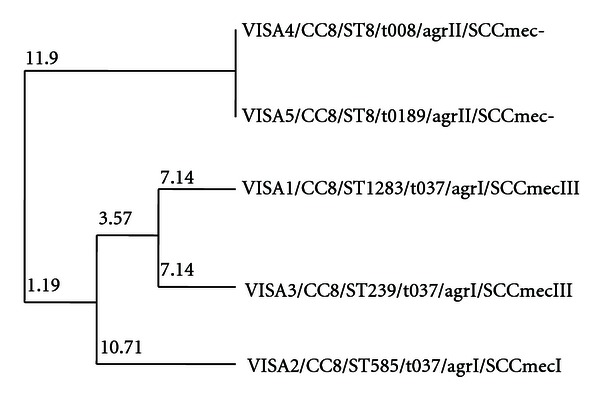
Dendrogram comparing sequence types (STs) of VISA isolates.

All of our MRSA-VISA isolates (two isolates from Isfahan in center of Iran and one isolate from Mashhad in northeast of Iran) had *spa* t037 and their STs were ST1283, ST585, and ST239 for VISA 1, 2, and 3, respectively. ST1283 and ST585 are single-locus variants (SLV) of ST239, the endemic strain of Asia (Figure 1) which our data suggest to be the probable common ancestor of our MRSA-VISA isolates. All of our five VISA isolates belonged to clonal complex 8 (CC8) that is the major endemic *S. aureus* clone (particularly MRSA) in Asia and also is one of the pandemic MRSA clones [33, 39–44]. 

Increasing antibiotic resistance in major *S. aureus* clones intensifies precautionary policies for public health care systems. In this study, we have shown the upward trend of MSSA- and MRSA-VISA clones in Iran. Moreover, findings of our study suggest that the isolated VISA strains most probably belong to endemic Asian clones. In addition, these data suggest that vancomycin resistance has the potential to become a widespread problem in both MRSA and MSSA strains.

## Figures and Tables

**Table 1:** Genetic characteristics of VISA isolates.

	*agr *	*Sccmec *	*pvl *	*hla *	*etc *	*ts1t *	*mecA *	*vanA *	* spa * type	*spa* repeat profile	ST	CC	MLST allelic profile
VISA1	I	III	+	+	+	+	+	−	t037	15-12-16-02-25-17-24	ST-1283	8	2-3-1-88-4-4-3
VISA2	I	I	−	+	−	−	+	−	t037	15-12-16-02-25-17-24	ST-585	8	2-1-1-1-4-4-3
VISA3	I	III	−	+	−	−	+	−	t037	15-12-16-02-25-17-24	ST-239	8	2-3-1-1-4-4-3
VISA4	II	−	+	+	−	−	−	−	t008	11-19-12-21-17-34-24-34-22-25	ST-8	8	3-3-1-1-4-4-3
VISA5	II	−	−	−	−	+	−	−	t0189	07-23-12-21-17-34	ST-8	8	3-3-1-1-4-4-3

*pvl*: Panton-Valentin Leukocidin, *sec*: Enterotoxin C, *tst1*: Toxic Shock Syndrome Toxin1, *hla*: alpha Hemolysin, *agr*: accessory gene regulator, *SCCmec*: Staphylococcal Cassette Chromosome, *spa*: Staphylococcal protein A.

**Table 2:** Phenotypic characteristics of VISA isolates.

	Gender/age	city	Sample/ward	Oxa	Van	Min	Lev	Cip	Tet	Cot	Gen	Cli	Rif	Oxa MIC	Van MIC	Van Agar screen
VISA1	M/15	Mashhad	Blood/neurology	R	S	S	S	S	R	S	R	S	S	128	8	+
VISA2	M/39	Isfahan	Throat/out patient	R	S	S	R	R	R	R	R	R	R	128	4	+
VISA3	M/50	Isfahan	Blood/neurology	R	S	R	S	S	S	S	S	R	R	64	4	+
VISA4	M/52	Mashhad	Wound/urgent	S	S	R	S	S	S	S	S	R	S	2	4	−
VISA5	M/71	Mashhad	Throat/Internal	S	S	I	R	R	R	R	S	R	I	2	4	+

Oxa: oxacillin, Van: vancomycin, Min: minocyclin, Lev: levofloxacin, Cip: ciprofloxacin, Tet: tetracycline, Cot: cotrimoxazole, Gen: gentamycin, Cli: clindamycin, Rif: rifampicin, R: resistant, S: susceptible.

**Table 3:** Primers used in this study.

Target	Primer	Sequence (5′-3′)	Product size (bp)	Reference
*mecA *	F	AGAAGATGGTATGTGGAAGTTAG	583	This study
	R	ATGTATGTGCGATTGTATTGC		

*pvl *	F	GGAAACATTTATTCTGGCTATAC	502	This study
	R	CTGGATTGAAGTTACCTCTGG		

*hla *	F	CGGTACTACAGATATTGGAAGC	744	This study
	R	TGGTAATCATCACGAACTCG		

*sec *	F	GGGAATGTTGGATGAAGG	900	This study
	R	AGGCAAGCACCGAAGTAC		

*tst1 *	F	TTATCGTAAGCCCTTTGTTG	398	This study
	R	TAAAGGTAGTTCTATTGGAGTAGG		

*agr *	Pan F	ATGCACATGGTGCACATGC		[26]
	R I	GTCACAAGTACTATAAGCTGCGAT	439	
	R II	GTATTACTAATTGAAAAGTGCCATAGC	572	
	R III	CTGTTGAAAAAGTCAACTAAAAGCTC	406	
	R IV	CGATAATGCCGTAATACCCG	657	

*SCCmec *	F *β*	ATTGCCTTGATAATAGCCYTCT	937	[25]
	R *α*3	TAAAGGCATCAATGCACAAACACT		
	F ccrC	CGTCTATTACAAGATGTTAAGGATAAT	518	
	R ccrC	CCTTTATAGACTGGATTATTCAAAATAT		
	F 1272	GCCACTCATAACATATGGAA	415	
	R 1272	CATCCGAGTGAAACCCAAA		
	F 5R*mecA *	TATACCAAACCCGACAACTAC	359	
	R 5R431	CGGCTACAGTGATAACATCC		
